# Indirect Effects of Executive Planning Functions and Affectivity on the Work Ethic of University Students

**DOI:** 10.3390/jintelligence13020020

**Published:** 2025-02-11

**Authors:** Jorge Vergara-Morales, Milenko Del Valle, Nancy Lepe

**Affiliations:** 1 Escuela de Psicología, Facultad de Salud y Ciencias Sociales, Universidad de Las Américas, Campus Chacabuco, Concepción CP 4030000, Chile; 2 Departamento de Ciencias Sociales, Facultad de Ciencias Sociales, Artes y Humanidades, Universidad de Antofagasta, Campus Coloso, Antofagasta CP 1240000, Chile; mdelvalle@uantof.cl; 3 Departamento de Diversidad e Inclusividad Educativa, Facultad de Ciencias de la Educación, Universidad Católica del Maule, Campus San Miguel, Talca CP 3460000, Chile; nlepe@ucm.cl

**Keywords:** work ethic, affectivity, executive functions, moral intuition

## Abstract

Work ethic represents a key factor for professional performance, as it guides behaviors relevant to the transparency and quality of work practices. Although a wide field of study has been developed, less research has analyzed the indirect influence of affective and cognitive factors involved in work ethic. Therefore, this study aims to assess the indirect effects of executive planning functions and affectivity on the work ethic of Chilean university students. The purpose is to test the following hypotheses: (1) executive planning functions have an indirect effect on work ethic through moral reasoning; (2) affectivity has an indirect effect on work ethic through moral intuition. The participants were a total of 582 Chilean university students from a university in the north (38.5%), one in the center (35.9%), and one in the southern area (25.6%). The data were analyzed using descriptive statistics, structural equation models (SEMs), and SEM mediation analysis. The results show the direct effect of moral reasoning (β = 0.47, *p* < .01) and moral intuition (β = 0.85, *p* < .01) on work ethic. Furthermore, they support the indirect effect of executive planning functions (β = 0.06, *p* < .01) and affectivity (β = 0.46, *p* < .01) on work ethic. The model explains 98% of the variance of work ethic, highlighting the critical roles of moral reasoning and moral intuition as psychological mechanisms that intervene and drive the effect of cognitive and affective factors. Theoretical and practical implications for teaching–learning processes in higher education are discussed.

## 1. Introduction

Work ethic has been defined “as an attitudinal construct pertaining to work-related values” (Sharma and Rai 2015, p. 2). Constitutes a relevant aspect of society, as it is essential to guide key organizational behaviors, such as job performance, quality, and productivity (Grabowski et al. 2019; Pourmofrad and Mohammadian 2017). The background shows that high levels of work ethic allow for mitigating and regulating deviant and ineffective behaviors in the workplace (Elliethey et al. 2024). On the one hand, its influence on developing problem-solving skills that facilitate good performance is highlighted (Sulistiowati et al. 2018). On the other hand, it is emphasized as a social skill aimed at shaping attitudes and behaviors in the workplace (Panigrahi and Al-Nashash 2019). Thus, it is argued that work ethic is a priority for organizations, as it can affect their performance and effectiveness (Salahudin et al. 2016; Banister 2017; Bataineh 2020; Benedicto and Caelian 2021). In the field of higher education, research suggests the importance of generating practical guidelines that guide behavior toward work ethic since when correct work values are not internalized, behaviors are promoted that entail negative consequences at a personal and institutional level. Because work ethic develops through a gradual process marked by personal experiences and the influence of outstanding people and significant figures (Grabowski et al. 2021), higher education institutions have an essential role in stimulating their teaching through appreciation, commitment, and respect for the profession (Harkriskayani and Putra 2024). This area of training involves the challenge and responsibility of “providing society with professionally prepared people with the criteria to develop their professional practice” (Gaete Quezada 2020, p. 2), which is strongly linked to the need to develop training processes teaching that articulate the guidelines of science and technical with the ethical principles that characterize the professions.

The responsibility of higher education institutions implies assuming the commitment to create social spaces necessary to respond to current social problems through a constant and systematic update of scientific knowledge that integrates the fundamental ethical principles for the orientation and modeling of learning (Méndez-Medrano et al. 2018). Although the background shows research on cognitive and affective processes that influence ethical decision-making, most studies focus on children and adolescents, with fewer on the adult population. Furthermore, most studies focus on general ethics, with fewer focused on specific domains such as work ethic. Also, most studies focus on measuring ethics based on moral dilemmas, with fewer exploring attitudes or dispositions to ethics in a specific field such as work. Finally, few studies analyze the direct influence of cognitive and affective processes on work ethic. In this regard, a few studies evaluate the indirect influence of these factors relevant to ethics in the professional field. That is, fewer studies analyze the intervening variables through which cognitive and affective factors exert their influence.

## 2. Literature Review

### 2.1. Work Ethic

From a psychological perspective, work ethic is configured by attitudes, beliefs, and emotional components that support the centrality of work in daily life activities (Grabowski et al. 2021). A high work ethic is based on the conviction that it represents a central value in life, which is why it must also be applied correctly in the workplace (Sharma and Rai 2015). For example, when honesty is emphasized during interactions with colleagues, it is assumed that honest behaviors should be central in the working spaces. Thus, work ethic can be understood as a collection of beliefs and motivations that reflect and support the elemental importance of work. (Sakr et al. 2022). Moreover, it can be considered normative guidance based on professional duties and rights, including awareness of responsibility for accomplishing the activities. In summary, it can be defined as a “system of attitudes and, secondly, a system of convictions (belief system) [constituted by] cognitive, emotional and behavioral components” (Czerw and Grabowski 2015, p. 505) that promote the importance and meaning of work. In the field of higher education, it is proposed that work ethic is related to professional ethics. Both concepts are interdependent. While work ethic prioritizes and reveals the values and virtues of all professions, professional ethics focuses on the fundamental values of work (Sakr et al. 2022). In this regard, moral reasoning constitutes a cognitive process that involves the evaluation of principles for decision-making.

### 2.2. Moral Reasoning

Moral reasoning refers to the structured process of thinking that analyzes and assesses from ethical principles (Bucciarelli et al. 2008). Regarding the configuration of moral reasoning, one of the most influential orientations corresponds to the cognitive perspective that states that moral judgment “is expressed in a theoretical structure organized into three levels—pre-conventional, conventional and post-conventional—[where the] levels and their stages represent structures of judgment and differentiated sociomoral perspectives that have cognitive development as a necessary, but not sufficient, condition” (Barba and Romo 2005, p. 69). Regarding the levels of moral development, the pre-conventional level refers to the individualistic perspective oriented from one’s interest to avoid punishments or obtain rewards. The conventional level corresponds to the perspective that implies considering oneself a member of society and is based on concordance and order. Finally, the post-conventional level refers to the autonomous perspective of reasoning based on universal ethical principles and values.

In summary, it can be stated that “moral judgments are derived from the application of conscious reasoning. Moral development across the lifespan reflects a growing capacity to articulate compelling reasons when making moral judgments” (Loewe 2017, p. 246). Therefore, moral judgment implies a process of moral growth that occurs progressively through levels of development, allowing for promoting and strengthening a superior capacity to value what is fair, directly influencing the configuration of ethical behavior (Ferguson et al. 2021; Tervo-Clemmens et al. 2023). From a neurophysiological perspective, it has been observed that the activation of the ventromedial prefrontal cortex facilitates the integration of information about results and beliefs during moral reasoning (Boccia et al. 2017). Based on the background reviewed, this research proposes that moral reasoning directly and positively influences the work ethic. Moreover, one of the cognitive processes related to moral reasoning refers to executive functions, through which it is possible to observe the degree of maturity of moral reasoning (Vera-Estay et al. 2016). Executive functions involve multiple skills related to the regulation of thought and behavior. The background suggests that executive functions impairments in childhood and adulthood are associated with poor social competence and psychopathological disorders that affect social adaptation (Vera-Estay et al. 2014). Studies with healthy, educated children and adults show that better executive skills contribute to more mature moral reasoning (Caporaso and Marcovitch 2024; Travis et al. 2011).

### 2.3. Executive Functions

Executive functions are defined as “a set of different and interdependent mental processes that control cognition and the regulation of behavior, such as problem-solving, planning, working memory, decision-making, monitoring, and inhibitory control” (Tamayo-Lopera et al. 2020, p. 22). According to the brain’s anatomical structure, the basis of executive functions is in the prefrontal cortex, composed of particular circuits and structures that activate working memory processes, action planning, mental flexibility, inhibitory control of automatic responses, verbal fluency, and decision-making (Jiménez-Puig et al. 2019). Thus, executive functions can be understood as self-regulation toward achieving goals since they promote cognitive control that guides and coordinates behavior adaptation to new situations beyond habitual and autonomous behaviors. In the educational context, it has been identified that “academic success […] depends largely on skills to organize, plan and prioritize time, select materials and available information, to separate fundamental ideas from accessory ones, to change the direction of an activity flexibly” (Gutiérrez-García and Landeros-Velázquez 2017, p. 399).

Concerning moral development, studies show that executive functions are positively related to moral reasoning since conceptual reasoning, cognitive flexibility, verbal fluency, and the use of feedback act as predictors of the maturity of moral reasoning (Vera-Estay et al. 2016). Furthermore, the study conducted by Piryaei et al. (2017) shows that executive planning functions positively influence the decision-making process, as planning involves a constructive-level resource that organizes responses that satisfy the constraints regarding taking a position.

In this regard, cognitive planning consists of structuring a plan to achieve a goal in an organized, strategic, and efficient manner. This mental ability allows for us to foresee how our actions can influence our current situation and guide us toward a different future state. In this context, it involves creating a mental representation of a goal and our current behavioral state about that goal (Domic-Siede et al. 2022). As moral judgment is crucial to configuring ethical behavior (Jiménez-Puig et al. 2019), this research proposes that executive planning functions will indirectly influence work ethic through moral reasoning. That is, moral reasoning would act as a psychological mechanism that drives the effect of executive planning functions toward work ethic. On the other hand, the background suggests that affective processes participate simultaneously with cognitive processes during decision-making, which indicates the relevance of moral intuition.

### 2.4. Moral Intuition

The perspective of moral intuition arises as a response to the rational models that have dominated the field of moral development, from which intuitive processing is proposed that is characterized by its “speed, automaticity, inaccessibility, parallel processing, and dependence on the context” (Quiroga 2013, p. 181). Regarding the development of moral judgment, it is indicated that moral positions can arise from the intuition that is “culturally driven, that is, they consist of rapid, involuntary and automatic responses driven by culturally and socially acquired principles […] and that […] are the product of a moral module innately programmed in the brain” (Dellantonio and Job 2012, p. 240). More specifically, intuition can be defined as “the sudden appearance in consciousness of a moral judgment, which includes an affective valence […] without any conscious reasoning of having gone through paths of search, weighing of evidence, or inference of conclusions” (Haidt 2001, p. 818). According to the anatomical structure of the brain, activation of the ventromedial prefrontal cortex and limbic regions such as the amygdala, temporal poles, and posterior cingulate cortex have been observed to influence the configuration of moral judgment driven by intuitive responses based on affective valence (Prehn et al. 2015). Based on the reviewed background, it is suggested that moral intuition will directly influence work ethics. Furthermore, considering that affective valence influences the configuration of intuitive processing, it is proposed that affectivity will directly influence moral intuition. In this sense, affectivity is understood to promote information processing towards ethical decision-making. By interpreting more complex expressions of thoughts from affective evaluations, comparing and integrating positive and negative feelings is possible instead of making sense of many conflicting logical reasons. One of the main characteristics of the intuitive decision-making system is its affective basis (Laing et al. 2022; Slovic and Västfjäll 2010).

### 2.5. Affectivity

Affectivity is defined as “a psychological construct that refers to mental states that involve evaluative feelings (e.g., feeling good-bad, pleasant-unpleasant situations)” (Díaz-García et al. 2020, p. 1). It is configured through a basic two-dimensional structure: (a) positive affect, referring to “the point to which a person feels enthusiastic, active, alert, with energy and rewarding participation” (Balladares and Saiz 2015, p. 65), and (b) negative affect, “which includes unpleasant emotions and feelings such as sadness, fear, anxiety, depression, boredom, and pessimism” (Osti and Porto 2015, p. 197). From the neuro-physiological perspective, affective valence (positive/negative) not only refers to an inherent quality of any external stimulus but also implies the activation of the limbic systems of the brain that generates hedonic reactions such as pleasure or disgust and motivational reactions such as reward-seeking or threat avoidance. The differences between positive and negative affectivity are explained through the hypothesis of affective modules, from which it is proposed that a “given neural module may not be permanently dedicated to a single affective function, but rather has multiple affective modes neurobiological as states or conditions change, which gives it different functions related to affective valence” (Berridge 2019, p. 226).

Therefore, affective modes imply the possibility of change in internal physiological states that produce modification in neurophysiological states that alter affective function as conditions change, allowing for the same limbic circuits to be activated by stimuli associated with positive and negative valence. In the educational field, it has been observed that positive affectivity predicts high levels of self-efficacy and academic performance since it allows for broadening attention focus, facilitating working memory and problem-solving, which leads to better responses that promote learning. In the field of moral development, studies show that affectivity influences the configuration of moral judgments since it assesses moral thinking. More specifically, positive affectivity promotes the growth of ethical thinking, and negative affectivity inhibits the development of thinking, facilitating the development of innate responses (Pastötter et al. 2013). Thus, the research proposes that affectivity indirectly influences the work ethic through moral intuition. Moreover, considering that affective valence influences the configuration of intuitive processing, affectivity is proposed to directly influence moral intuition. Furthermore, the indirect effect of affectivity on work ethics is proposed, which shows the relevance of affective processes in the ethical field.

## 3. The Present Study

**Figure 1:**
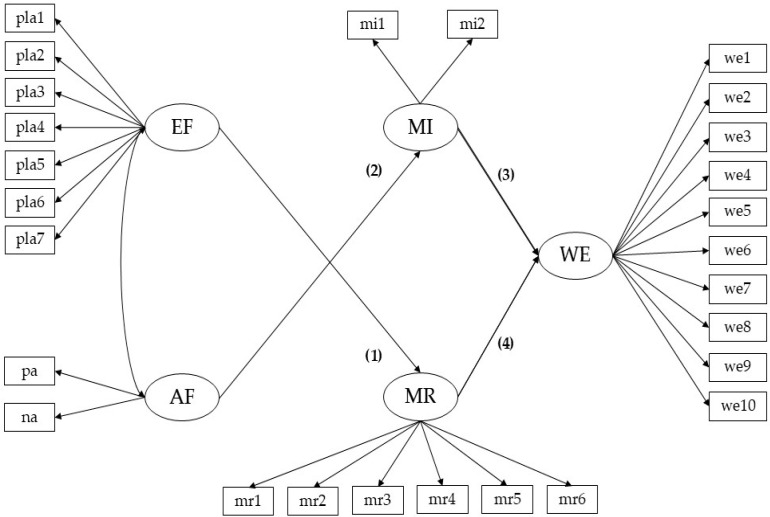
Hypothetical SEM model. AF = affectivity; EF = executive planning functions; MI = moral intuition; MR = moral reasoning; WE = work ethic.

Based on the review of the background, the estimation of the following hypothetical model is proposed: (1) executive planning functions have a positive effect on moral reasoning, (2) affectivity has a positive effect on moral intuition, (3) moral intuition positively affects work ethic, and (4) moral reasoning positively affects work ethic (see Figure 1). Based on the hypothetical model, this study aims to test the following hypotheses: (H1) executive planning functions indirectly affect work ethic through moral reasoning; (H2) affectivity indirectly influences work ethic through moral intuition. This study aims to assess the indirect effects of executive planning functions and the affectivity on the work ethic of Chilean university students.

## 4. Materials and Methods

### 4.1. Design

This study was carried out using a quantitative, cross-sectional, and non-experimental design. An explanatory associative strategy was used since a hypothetical model composed of relationships of variables that are derived from an underlying theory was evaluated (Ato et al. 2013).

### 4.2. Participants

The participants were a total of 582 Chilean university students coming from a university in the north (38.5%), one from the center (35.9%), and another from the south of Chile (25.6%); 77.7% were of the female gender (n = 452), 22% were of the male gender (n = 128), and 0.3% indicated another gender (n = 2). Participants ranged from 20 to 29 years, averaging 22.23 years (SD = 1.77 years). Participants were selected through intentional convenience sampling, considering intact cohorts. This selection process facilitated student access since data collection was conducted with the least interference in academic activities. However, one disadvantage of the sampling procedure is the incorporation of biases, such as those observed regarding gender, which is included as a study limitation.

### 4.3. Instruments

#### 4.3.1. Positive and Negative Affectivity Scale (PANAS)

The instrument is composed of a total of 20 items that are distributed in two factors, (a) positive affectivity (10 items) and (b) negative affectivity (10 items), which describe adjectives associated with habitual feelings and emotions. The items were answered on a Likert scale, with response options ranging from 1 (not at all) to 7 (extremely). The instrument has presented adequate levels of reliability (α > 0.88) and validity (χ^2^ = 367.11, *p* < 0.01; CFI = 0.92; TLI = 0.90; RMSEA = 0.06) (López-Gómez et al. 2015). The mean scores of positive and negative affectivity were used to construct the latent variable of affectivity.

#### 4.3.2. EFECO Scale

The instrument is composed of a total of 67 items that measure different executive functions following the measurement model proposed by García-Gómez (2015), from which the following factors are considered: (a) monitoring; (b) inhibition; (c) cognitive flexibility; (d) emotional control; (e) planning; (f) organization of materials; (g) initiative; (h) working memory. For the case of the study, the items referring to the planning factor were considered (7 items, e.g., “I find it difficult to think or plan things in advance”). The items were answered on a Likert scale, with responses ranging from 1 (never) to 7 (always). The scale has shown adequate levels of internal consistency (α = 0.64–0.95) and validity (χ^2^ = 38.87, CFI = 0.98, RMSEA = 0.06 [0.04–0.09], SRMR = 0.03) (Ramos-Galarza et al. 2019). The seven items that measure executive planning functions were used to construct the latent variable.

#### 4.3.3. The Moral Development Scale for Professionals (MDSP)

The instrument is composed of a total of 12 items (e.g., “Important to listen to what people mean in moral issues”) that are distributed in three factors that measure the conventional and post-conventional stages of Kohlberg’s moral development perspective. In the case of this research, the items associated with the post-conventional factor of moral development were considered since they represent the stage at which university students are located. The items were answered on a Likert scale ranging from 1 (strongly disagree) to 7 (strongly agree). The instrument has presented a measurement model with adequate levels of reliability (α = 0.64) and validity (χ^2^ = 81.12, *p* < 0.01; RMSEA = 0.04) (Söderhamn et al. 2011). The six items that measure post-conventional stage were used to construct the latent variable.

#### 4.3.4. Moral Foundations Questionnaire (MFQ)

The instrument is oriented to measure five innate moral foundations: (a) care/harm, (b) justice/injustice, (c) loyalty/betrayal, (d) authority/subversion, and (e) purity/degradation. The first part of the questionnaire examines the degree of relevance of moral foundations. This section consists of 11 items answered on a 7-point Likert scale (1 = not at all relevant; 7 = completely relevant). The second part studies the degree of agreement or disagreement with the moral foundations. It is composed of 11 items that were answered on a 7-point Likert scale (1 = totally disagree, 7 = totally agree). The instrument has presented adequate levels of reliability (α = 0.84), and convergent and divergent validity based on correlations with instruments intended to measure empathy, values, and attitudes toward topics such as religion or politics (Miranda-Rodríguez and García-Méndez 2019). The mean scores of the relevance of moral foundations and degree of agreement with moral foundations were used to construct the latent variable of moral intuition.

#### 4.3.5. The Work Ethic Scale (TWES)

The instrument is composed of 10 items that constitute a single factor that measures the ethical disposition towards work in the professional field, considering work from the central interest in life, morality, and intrinsic motivation. Items were answered on a Likert scale that ranged from 1 (completely disagree) to 7 (completely agree). The instrument has presented an unifactorial structure with adequate levels of reliability (α = 0.87) and validity (CFI = 0.95; TLI = 0.93; RMSEA = 0.07) and reliability (α = 0.87) (Sharma and Rai 2015). The 10 items that measure work ethic were used to construct the latent variable.

### 4.4. Procedure

This study was approved by the Scientific Research Ethics Committee of the Central University of Chile (resolution 52/2022). The questionnaires were administered in person in the classroom, under the supervision of a trained research assistant. The students signed a letter of informed consent. This research was guided by the ethical considerations of the American Psychological Association (APA).

### 4.5. Data Analyses

Descriptive analysis was carried out by calculating the mean and standard deviation and measuring skewness and kurtosis. The internal consistency of the data was evaluated using Cronbach’s alpha coefficient, considering a lower limit of 0.60 to identify acceptable reliability (Haynes et al. 1995). The hypothesized model was fitted to the observed data using the weighted least squares with mean (WLSM) estimation method. The goodness of fit of the measurement model was assessed considering the following indices and criteria: (a) χ^2^/df: a good fit is indicated by values <5 (Diamantopoulos and Siguaw 2000); (b) comparative fit index (CFI) and Tucker–Lewis index (TLI): an acceptable fit is indicated by values ≥0.90, and a good fit is determined by values ≥0.95; (c) root mean square error of approximation (RMSEA): an acceptable fit is determined by values ≤0.08 (90% CI ≤ 0.10), and a good fit is indicated by values ≤0.06 (90% CI ≤ 0.08) (Kelloway 2015). Finally, the indirect effects were considered statistically significant if the 95% confidence interval estimates did not pass through the value zero (Hayes 2018).

## 5. Results

### 5.1. Descriptive Statistics and Reliability Coefficients

Table 1 shows that the mean scores of all variables present a favorable trend since they were higher than the theoretical mean (MT = 4.00). The highest mean score is observed in work ethic (M = 5.44), followed by the degree of agreement with moral foundations (M = 5.09) and positive affectivity (M = 5.06). On the other hand, it is observed that the mean scores present adequate levels of dispersion since the standard deviation values were close to 1. Moreover, the skewness and kurtosis values allow for it to infer a tendency towards univariate normality since the values obtained were less than ±2 (George and Mallery 2010). Finally, the values of the α coefficient show that the observed data present adequate levels of reliability.

### 5.2. SEM Analysis

**Figure 2:**
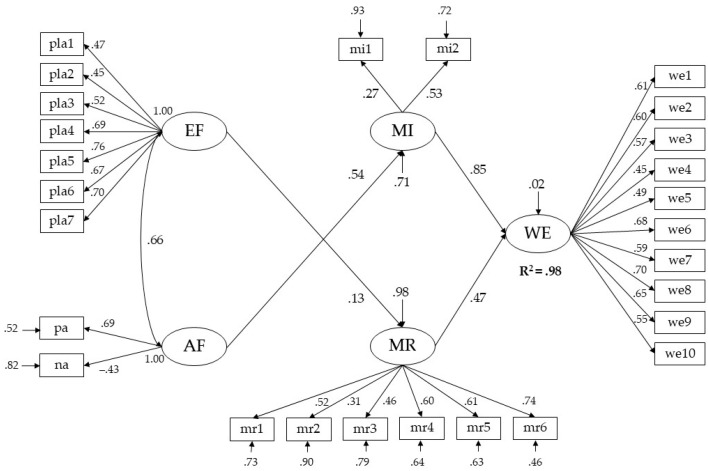
Hypothesized SEM model (standardized coefficients). AF = affectivity; EF = executive planning functions; MI = moral intuition; MR = moral reasoning; WE = work ethic.

The results of the assessment of the hypothetical model allow for it to accept the fit of the model to the observed data since the values of the goodness of fit are within the recommended limits (χ^2^/df = 4.26; CFI = 0.91; TLI = 0.90; RMSEA = 0.075; SRMR = 0.06). The items of the model were correlated with the latent variables they measured since the factor loadings presented values equal to or greater than 0.30. Furthermore, they were statistically significant at a *p* < .01 level. Regarding the model’s relationships, the positive and direct effect of executive planning functions on moral reasoning is observed (β = 0.13, *p* < .01), as well as the positive and direct effect of affectivity on moral intuition (β = 0.54, *p* < .01). In this regard, it is inferred that as executive planning functions increase, so does moral reasoning. Furthermore, as affectivity increases, moral intuition also increases. However, the effect of executive planning functions on moral reasoning is smaller than the effect of affectivity on moral intuition, where the standardized beta value indicates that an increase of 1 standard deviation of affectivity is associated with an increase of 0.54 standard deviations in work ethic. Finally, only moral reasoning (β = 0.47, *p* < .01) and moral intuition (β = 0.85, *p* < .01) had a positive and direct effect on work ethic. In this regard, it is observed that moral intuition is the most significant predictor of work ethic since the standardized beta value indicates that an increase of 1 standard deviation is associated with an increase of 0.85 standard deviations in work ethic. The model’s relationships explain 98% of the variance in work ethic (see Figure 2).

### 5.3. SEM Mediation Analysis

Based on the relationships confirmed in the hypothetical model, Table 2 shows the assessment results of the indirect effect of executive planning functions on the work ethic. In this regard, a positive and significant indirect effect is observed (β = 0.06, *p* < .01) through moral reasoning. Because the estimates of the confidence intervals do not pass through the value zero, the significance of the indirect effect is confirmed.

Table 3 shows the assessment results of the indirect effect of affectivity on the work ethic. In this regard, a positive and significant indirect effect is observed (β = 0.46, *p* < .01) through moral intuition. Because the estimates of the confidence intervals do not pass through the value zero, the significance of the indirect effect is confirmed.

## 6. Discussion

This study aims to evaluate the indirect effects of executive planning functions and affectivity on the work ethic of Chilean university students. The results show that the most significant relationships were between moderate and high. Regarding the cognitive dimension, executive planning functions positively affected moral reasoning, and only moral reasoning had a positive effect on work ethic. Regarding the affective factors, affectivity positively affected moral intuition, and only moral intuition had a positive effect on work ethic. Regarding the research hypotheses, the indirect effect of executive planning functions and affectivity on work ethic was verified. From the findings, it is inferred that when university students perceive cognitive planning skills and affectivity favorably, the activation of attitudes, beliefs, and emotions that support the centrality of work in professional activities is promoted. These results are consistent with research findings that have observed that high levels of executive functions demonstrate high levels of moral reasoning, which implies high degrees of maturity for ethical decision-making (Boccia et al. 2017; Vera-Estay et al. 2016).

In the case of this study, it is observed that when students activate their resources to organize responses that satisfy the constraints for assuming an ethical position, high levels of moral reasoning are promoted in the workplace. Moreover, it is inferred that moral reasoning constitutes a psychological mechanism that drives the positive effect of executive planning functions toward work ethics. That is, moral reasoning gives direction and meaning to the influence of executive functions on work ethics. On the other hand, it is observed that the results coincide with the findings of a study showing that university students who value moral thinking positively show high levels of moral intuition associated with high levels of integration of sociocultural factors that constitute innate foundations that affect ethical decision-making (Pastötter et al. 2013). In this regard, moral intuition is a psychological mechanism that provides direction and meaning to the influence of affectivity on work ethics. These results coincide with the findings that show work ethics is related, on the one hand, to affective aspects associated with positive feelings for ethical behavior and, on the other hand, to cognitive aspects related to behavioral planning for the pursuit of work success (Grabowski et al. 2021).

Based on the findings, it is concluded that executive planning functions and affectivity constitute two types of processing that positively and indirectly affect work ethics. This influence is generated through moral reasoning, which guides and directs the influence of cognitive processing. Furthermore, it occurs through moral intuition, which guides and directs the influence of affective processing. In this way, both moral reasoning and moral intuition are essential to intervene and guide the relationships of influence of cognitive and affective resources in the field of work ethics. From the perspective of professional training, higher education institutions must promote innovative and ethical programs that consider strengthening cognitive and affective skills that facilitate the adequate development of ethics at work. This implies the challenge of creating teaching guidelines that integrate comprehensive, metacognitive, and self-regulation aspects, which promote the activation of cognitive and affective processing for ethical decision-making in the workplace. In this sense, universities can encourage learning experiences on moral reasoning and moral intuition based on integrating training workshops into the curriculum. This process must consider the implementation of innovative teaching methodologies that facilitate interest and satisfaction in developing cognitive and affective skills that are fundamental for ethical decision-making (Morasse et al. 2021). Finally, this study highlights the critical role of moral reasoning and moral intuition in shaping college students’ professional ethics, providing a foundation for future research and practical applications in education and workforce development.

Regarding this study’s limitations, the use of a cross-sectional design does not allow for the knowledge of the relationships of the variables over time, so future research should consider the use of longitudinal designs, as they will increase the evidence of the modeled relationships. Another limitation of the design is that it does not allow for strong causal statements compared to a longitudinal study, which reinforces the recommendation for longitudinal designs. Moreover, self-reporting represents a limitation, so it is suggested that future research includes behavioral activities to measure the variables analyzed, such as task performance simulations that validate self-reported measures of work ethic. On the other hand, the gender composition of the sample represents another limitation, so it is suggested that future research consider levels of balance in the gender distribution of the selected samples. Finally, another limitation was that only executive planning functions were measured. Therefore, future research must include other executive functions in the analysis.

## Figures and Tables

**Table 1:** Descriptive and reliability analysis.

Variables	M	SD	Skewness	Kurtosis	α
PA	5.06	0.84	−0.40	−0.04	0.85
NA	3.34	1.05	0.27	−0.31	0.86
PLA	4.77	1.03	−0.42	0.18	0.79
MI1	4.45	0.99	−0.07	−0.09	0.82
MI2	5.09	0.69	−0.05	0.19	0.67
MR	4.69	0.93	−0.59	0.47	0.71
WE	5.44	0.83	−0.49	−0.18	0.79

PA = positive affectivity; NA = negative affectivity; PLA = executive planning functions; MI1 = relevance of moral foundations; MI2 = degree of agreement with moral foundations; MR = moral reasoning; WE = work ethic.

**Table 2:** The indirect effect of the executive functions.

Effect of EF to	WE	IC95%
Direct	0.00	-
Indirect (via MR)	0.06 **	0.02–0.11
Total	0.06 **	0.02–0.11

EF = executive planning functions; WE = work ethic; MR = moral reasoning; ** *p* < 0.01.

**Table 3:** The indirect effect of the affectivity.

Effect of AF to	WE	IC95%
Direct	0.00	-
Indirect (via MI)	0.46 **	0.38–0.54
Total	0.46 **	0.38–0.54

AF = affectivity; WE = work ethic; MI = moral intuition; ** *p* < 0.01.

## Data Availability

The datasets used in this manuscript are not readily available because the data are part of ongoing research. Requests to access the datasets should be directed to jvergaram@udla.cl.

## References

[B1-jintelligence-13-00020] Ato Manuel, López Javier, Benavente Ana (2013). A classification system for research designs in psychology. Anales de Psicología/Annals of Psychology.

[B2-jintelligence-13-00020] Balladares Silvia, Saiz Mario (2015). Sentimiento y afecto. Ciencias Psicológicas.

[B3-jintelligence-13-00020] Banister Christina (2017). Work Ethic, Turnover, and Performance: An Examination of Predictive Validity for Entry-Level Employees. Ph.D. thesis.

[B4-jintelligence-13-00020] Barba Bonifacio, Romo José M. (2005). Desarrollo del juicio moral en la educación superior. Revista Mexicana de Investigación Educativa.

[B5-jintelligence-13-00020] Bataineh Mohammad T. (2020). The Effect of Work Ethics on Job Performance in International SMEs in Al-Hassan Industrial Estate. International Review of Management and Marketing.

[B6-jintelligence-13-00020] Benedicto Helen, Caelian Merlita (2021). The Influence of Work Ethics on Job Performance of Government Employees. Philippine Social Science Journal.

[B7-jintelligence-13-00020] Berridge Kent C. (2019). Affective valence in the brain: Modules or modes? Nature reviews. Neuroscience.

[B8-jintelligence-13-00020] Boccia Maddalena, Dacquino Claudia, Piccardi Laura, Cordellieri Pierluigi, Guariglia Cecilia, Ferlazzo Fabio, Ferracuti Stefano, Giannini Anna M. (2017). Neural foundation of human moral reasoning: An ALE meta-analysis about the role of personal perspective. Brain Imaging and Behavior.

[B9-jintelligence-13-00020] Bucciarelli Monica, Khemlani Sangeet, Johnson-Laird Phil N. (2008). The psychology of moral reasoning. Judgment and Decision Making.

[B10-jintelligence-13-00020] Caporaso Jesica, Marcovitch Stuart (2024). Executive function as a mechanism for the emergence and expression of moral knowledge. Advances in Child Development and Behavior.

[B11-jintelligence-13-00020] Czerw Agnieszka, Grabowski Damian (2015). Work attitudes and work ethic as predictors of work engagement among Polish employees. Polish Psychological Bulletin.

[B12-jintelligence-13-00020] Dellantonio Sara, Job Remo, Magnani Lorenzo, Li Ping (2012). Moral Intuitions vs. Moral Reasoning. A Philosophical Analysis of the Explanatory Models Intuitionism Relies On. Philosophy and Cognitive Science. Studies in Applied Philosophy, Epistemology and Rational Ethics.

[B13-jintelligence-13-00020] Diamantopoulos Adamantios, Siguaw Judy A. (2000). Introducing LISREL. Introducing Statistical Methods.

[B14-jintelligence-13-00020] Díaz-García Amanda, González-Robles Alberto, Mor Sonia, Mira Adriana, Quero Soledad, García-Palacios Asucena, Baños Rosa M., Botella Cristina (2020). Positive and Negative Affect Schedule (PANAS): Psychometric properties of the online Spanish version in a clinical sample with emotional disorders. BMC Psychiatry.

[B15-jintelligence-13-00020] Domic-Siede Marcos, Irani Martín, Ramos-Henderson Miguel, Calderón Carlos, Ossandón Tomas, Perrone-Bertolotti Marcela (2022). La planificación cognitiva en el contexto de la evaluación neuropsicológica e investigación en neurociencia cognitiva: Una revisión sistemática. Terapia Psicológica.

[B16-jintelligence-13-00020] Elliethey Nancy S., Hashish Ebtsam Aly Abou, Elbassal Nariman Ahmed Mohamed (2024). Work ethics and its relationship with workplace ostracism and counterproductive work behaviours among nurses: A structural equation model. BMC Nursing.

[B17-jintelligence-13-00020] Ferguson Heather J., Brunsdon Victoria E. A., Bradford Elisabeth E. F. (2021). The developmental trajectories of executive function from adolescence to old age. Scientific Reports.

[B18-jintelligence-13-00020] Gaete Quezada Ricardo (2020). Management by values and social responsibility in Chilean state universities. Revista Digital de Investigación en Docencia Universitaria [Digital Journal of University Teaching Research].

[B19-jintelligence-13-00020] García-Gómez Andrés (2015). Desarrollo y validación de un cuestionario de observación para la evaluación de las funciones ejecutivas en la infancia. Revista Intercontinental de Psicología y Educación.

[B20-jintelligence-13-00020] George Darren, Mallery Paul (2010). SPSS for Windows Step by Step: A Simple Guide and Reference, 17.0 Update.

[B21-jintelligence-13-00020] Grabowski Damian, Chudzicka-Czupała Agata, Stapor Katarzyna (2021). Relationships between work ethic and motivation to work from the point of view of the self-determination theory. PLoS ONE.

[B22-jintelligence-13-00020] Grabowski Damian, Chudzicka-Czupała Agata, Chrupała-Pniak Małgorzata, Mello Abby L., Paruzel-Czachura Mariola (2019). Work ethic and organizational commitment as conditions of unethical pro-organizational behavior: Do engaged workers break the ethical rules?. International Journal of Selection and Assessment.

[B23-jintelligence-13-00020] Gutiérrez-García Ana G., Landeros-Velázquez María G. (2017). Evaluación de Funciones Ejecutivas en Estudiantes Universitarios con Niveles de Autoeficacia Percibida Baja. Revista Electrónica de Psicología Iztacala.

[B24-jintelligence-13-00020] Haidt Jonathan (2001). The emotional dog and its rational tail: A social intuitionist approach to moral judgment. Psychological Review.

[B25-jintelligence-13-00020] Harkriskayani Idah, Putra Purnama (2024). The Influence of Educational Background and Work Experience on Employee Work Ethic. Human Capital and Organizations (HCO).

[B26-jintelligence-13-00020] Hayes Andrew F. (2018). Introduction to Mediation, Moderation, and Conditional Process Analysis: A Regression-Based Approach (Methodology in the Social Sciences).

[B27-jintelligence-13-00020] Haynes Stephen N., Richard David C. S., Kubany Edward S. (1995). Content validity in psychological assessment: A functional approach to concepts and methods. Psychological Assessment.

[B28-jintelligence-13-00020] Jiménez-Puig Elizabeth, Broche-Pérez Yunier, Hernández-Caro Amarys A., Díaz-Falcón Dayana (2019). Funciones ejecutivas, cronotipo y rendimiento académico en estudiantes universitarios. Revista Cubana de Educación Superior.

[B29-jintelligence-13-00020] Kelloway E. Kevin (2015). Using Mplus for Structural Equation Modeling: A Researcher’s Guide.

[B30-jintelligence-13-00020] Laing Patrick, Davey Christopher G., Harrison Ben J. (2022). Influence of negative mood states on moral decision-making. Psychiatry Research Communications.

[B31-jintelligence-13-00020] Loewe Daniel (2017). Virtudes, racionalidad y el desarrollo moral. Alpha: Revista de Artes, Letras y Filosofía.

[B32-jintelligence-13-00020] López-Gómez Irene, Hervás Gonzalo, Vázquez Carmelo (2015). Adaptación de las “Escalas de afecto positivo y negativo” (PANAS) en una muestra general Española [An adaptation of the Positive and Negative Affect Schedules (PANAS) in a Spanish general sample]. Behavioral Psychology.

[B33-jintelligence-13-00020] Méndez-Medrano Cristian G., Torres-Gangotena Mario W., Camatón-Arizábal Segundo B. (2018). Importancia de la ética en la Educación Superior. Dominio de Las Ciencias.

[B34-jintelligence-13-00020] Miranda-Rodríguez Rubén A., García-Méndez Mirna (2019). Construcción de una Escala de Dominio Moral en Adolescentes. Revista Iberoamericana de Diagnóstico y Evaluación.

[B35-jintelligence-13-00020] Morasse Fréderick, Vera-Estay Evelyn, Beauchamp Miriam H. (2021). Using virtual reality to optimize assessment of sociomoral skills. Virtual Reality.

[B36-jintelligence-13-00020] Osti Andréia, Porto Ana P. (2015). Asociación entre afectos y optimismo en estudiantes del curso de Pedagogía. Revista Colombiana de Educación.

[B37-jintelligence-13-00020] Panigrahi Shrikant, Al-Nashash Hatem Mahmoud (2019). Quality Work Ethics and Job Satisfaction: An Empirical Analysis. QUALITY Access to Success.

[B38-jintelligence-13-00020] Pastötter Bernhard, Gleixner Sabine, Neuhauser Theresa, Bäuml Karl-Heinz H. (2013). To push or not to push? Affective influences on moral judgment depend on decision frame. Cognition.

[B39-jintelligence-13-00020] Piryaei Salehe, Ashkzari Molouk Khademi, Nejati Vahid, Arshadi Nasrin, Talkhabi Mahmoud (2017). Cognitive Functions and the Model of Decision-Making Competence: The Specific Case of Organizational Setting. International Journal of Behavioral Sciences.

[B40-jintelligence-13-00020] Pourmofrad Ahmad. R., Mohammadian Moghaddaseh (2017). Study the Effect of Ethical Principles on Job Commitment and Employees’ Satisfaction. International Review of Management and Marketing.

[B41-jintelligence-13-00020] Prehn Kristin, Korczykowski Marc, Rao Hengyi, Fang Zhuo, Detre John A., Robertson Diana C. (2015). Neural Correlates of Post-Conventional Moral Reasoning: A Voxel-Based Morphometry Study. PLoS ONE.

[B42-jintelligence-13-00020] Quiroga María P. (2013). El Innatismo Moral, un nuevo Paradigma de Desarrollo Moral, aportaciones desde la Cognición y la Neurociencia [The Moral Nativism, a new Paradigm of Moral Development, contributions from the Cognition and Neuroscience]. Acción Psicológica.

[B43-jintelligence-13-00020] Ramos-Galarza Carlos, Bolaños-Pasquel Mónica, García-Gómez Andrés, Martínez-Suárez Pedro, Jadán-Guerrero Janio (2019). La Escala EFECO para Valorar Funciones Ejecutivas en Formato de Auto-Reporte. Revista Iberoamericana de Diagnóstico y Evaluación.

[B44-jintelligence-13-00020] Sakr Fouad, Haddad Chadia, Zeenny Rony M., Sacre Hala, Akel Marwan, Iskandar Katia, Hajj Aline, Salameh Pascale (2022). Work Ethics and Ethical Attitudes among Healthcare Professionals: The Role of Leadership Skills in Determining Ethics Construct and Professional Behaviors. Healthcare.

[B45-jintelligence-13-00020] Salahudin Shahrul Nizam, Baharudin Siti Sarah, Abdullah Muhammad Safizal, Osman Abdullah (2016). The Effect of Islamic Work Ethics on Organizational Commitment. Procedia Economics and Finance.

[B46-jintelligence-13-00020] Sharma Baldev R., Rai Snigdha (2015). Study to Develop an Instrument to Measure Work Ethic. Global Business Review.

[B47-jintelligence-13-00020] Slovic Paul, Västfjäll Daniel (2010). Affect, moral intuition, and risk. Psychological Inquiry.

[B48-jintelligence-13-00020] Söderhamn Olle, Bjørnestad John O., Skisland Anne, Cliffordson Christina (2011). Construct validity of the Moral Development Scale for Professionals (MDSP). Journal of Multidisciplinary Healthcare.

[B49-jintelligence-13-00020] Sulistiowati Sulistiowati, Komari Nurul, Dhamayanti Endang (2018). The Effects of Person-Job Fit on Employee Engagement Among Lecturers in Higher Education Institutions: Is There a Difference Between Lecturers in Public and Private Higher Education Institutions?. International Review of Management and Marketing.

[B50-jintelligence-13-00020] Tamayo-Lopera Diego A., Hernández-Calle Jonathan A., Carrillo-Sierra Sandra, Hernández-Lalinde Juan (2020). Funciones ejecutivas tardías en estudiantes de undécimo grado de colegios oficiales de Cúcuta y Envigado, Colombia. AVFT.

[B51-jintelligence-13-00020] Tervo-Clemmens Brenden, Calabro Finnegan J., Parr Ashley C., Fedor Jennifer, Foran William, Luna Beatriz (2023). A canonical trajectory of executive function maturation from adolescence to adulthood. Nature Communications.

[B52-jintelligence-13-00020] Travis Frederick, Harung Harald S., Lagrosen Yvonne (2011). Moral development, executive functioning, peak experiences and brain patterns in professional and amateur classical musicians: Interpreted in light of a Unified Theory of Performance. Consciousness and Cognition.

[B53-jintelligence-13-00020] Vera-Estay Evelyn, Seni Anne G., Champagne Caroline, Beauchamp Miriam H. (2016). All for One: Contributions of Age, Socioeconomic Factors, Executive Functioning, and Social Cognition to Moral Reasoning in Childhood. Frontiers in Psychology.

[B54-jintelligence-13-00020] Vera-Estay Evelyn, Dooley Julian J., Beauchamp Miriam H. (2014). Cognitive underpinnings of moral reasoning in adolescence: The contribution of executive functions. Journal of Moral Education.

